# Methylation-based markers of aging and lifestyle-related factors and risk of breast cancer: a pooled analysis of four prospective studies

**DOI:** 10.1186/s13058-022-01554-8

**Published:** 2022-09-06

**Authors:** Pierre-Antoine Dugué, Clara Bodelon, Felicia F. Chung, Hannah R. Brewer, Srikant Ambatipudi, Joshua N. Sampson, Cyrille Cuenin, Veronique Chajès, Isabelle Romieu, Giovanni Fiorito, Carlotta Sacerdote, Vittorio Krogh, Salvatore Panico, Rosario Tumino, Paolo Vineis, Silvia Polidoro, Laura Baglietto, Dallas English, Gianluca Severi, Graham G. Giles, Roger L. Milne, Zdenko Herceg, Montserrat Garcia-Closas, James M. Flanagan, Melissa C. Southey

**Affiliations:** 1 Precision Medicine, School of Clinical Sciences at Monash Health, Monash University, Clayton, VIC Australia; 2 Cancer Epidemiology Division, Cancer Council Victoria, Melbourne, VIC Australia; 3 Centre for Epidemiology and Biostatistics, Melbourne School of Population and Global Health, The University of Melbourne, Parkville, VIC Australia; 4 Divison of Cancer Epidemiology and Genetics, National Cancer Institute, Bethesda, USA; 5 International Agency for Research On Cancer (IARC), Lyon, France; 6 Department of Medical Sciences, School of Medical and Life Sciences, Sunway University, Bandar Sunway, Malaysia; 7 Department of Surgery and Cancer, Faculty of Medicine, Imperial College London, London, UK; 8 AMCHSS, Sree Chitra Tirunal Institute for Medical Sciences and Technology, Thiruvananthapuram, Kerala India; 9 Department of Biomedical Sciences, University of Sassari, Sassari, Italy; 10 MRC Centre for Environment and Health, School of Public Health, Imperial College London, London, UK; 11 Unit of Cancer Epidemiology, Città della Salute e Della Scienza University-Hospital, Turin, Italy; 12 Department of Research, Epidemiology and Prevention Unit, Fondazione IRCCS Istituto Nazionale dei Tumori di Milano, Milan, MI Italy; 13 Dipartimento di Medicina Clinica e Chirurgia Federico II University, Naples, Italy; 14 Hyblean Association for Epidemiological Research AIRE-ONLUS, Ragusa, Italy; 15 Italian Institute for Genomic Medicine (IIGM), Turin, Italy; 16 Department of Clinical and Experimental Medicine, University of Pisa, 56126 Pisa, Italy; 17 CESP UMR1018, Paris-Saclay University, UVSQ, Inserm, Gustave Roussy, Villejuif, France; 18 Department of Clinical Pathology, Melbourne Medical School, The University of Melbourne, Parkville, VIC Australia

**Keywords:** Prospective study, DNA methylation, Epigenetic aging, Lifestyle, Breast cancer risk

## Abstract

**Background:**

DNA methylation in blood may reflect adverse exposures accumulated over the lifetime and could therefore provide potential improvements in the prediction of cancer risk. A substantial body of research has shown associations between epigenetic aging and risk of disease, including cancer. Here we aimed to study epigenetic measures of aging and lifestyle-related factors in association with risk of breast cancer.

**Methods:**

Using data from four prospective case–control studies nested in three cohorts of European ancestry participants, including a total of 1,655 breast cancer cases, we calculated three methylation-based measures of lifestyle factors (body mass index [BMI], tobacco smoking and alcohol consumption) and seven measures of epigenetic aging (Horvath-based, Hannum-based, *PhenoAge* and *GrimAge*). All measures were regression-adjusted for their respective risk factors and expressed per standard deviation (SD). Odds ratios (OR) and 95% confidence intervals (CI) were calculated using conditional or unconditional logistic regression and pooled using fixed-effects meta-analysis. Subgroup analyses were conducted by age at blood draw, time from blood sample to diagnosis, oestrogen receptor-positivity status and tumour stage.

**Results:**

None of the measures of epigenetic aging were associated with risk of breast cancer in the pooled analysis: Horvath ‘age acceleration’ (AA): OR per SD = 1.02, 95%CI: 0.95–1.10; AA-Hannum: OR = 1.03, 95%CI:0.95–1.12; *PhenoAge*: OR = 1.01, 95%CI: 0.94–1.09 and *GrimAge*: OR = 1.03, 95%CI: 0.94–1.12, in models adjusting for white blood cell proportions, body mass index, smoking and alcohol consumption. The BMI-adjusted predictor of BMI was associated with breast cancer risk, OR per SD = 1.09, 95%CI: 1.01–1.17. The results for the alcohol and smoking methylation-based predictors were consistent with a null association. Risk did not appear to substantially vary by age at blood draw, time to diagnosis or tumour characteristics.

**Conclusion:**

We found no evidence that methylation-based measures of aging, smoking or alcohol consumption were associated with risk of breast cancer. A methylation-based marker of BMI was associated with risk and may provide insights into the underlying associations between BMI and breast cancer.

**Supplementary Information:**

The online version contains supplementary material available at 10.1186/s13058-022-01554-8.

## Introduction

Numerous studies have investigated the association of blood DNA methylation and breast cancer risk, for example, at breast cancer-specific genes [1–3], and overall found mixed results [4]. Lower global levels of DNA methylation are thought to reflect genomic instability and have been hypothesised to increase the risk of cancer [5], but while several studies were conducted in the context of breast cancer [6–9] they together suggested that there is no substantial association [10]. At individual cytosine-guanine (CpG) sites, our meta-analysis of individual-participant data (1,663 incident cases and matched controls) from the Melbourne Collaborative Cohort Study (MCCS), the European Prospective Investigation into Cancer and Nutrition (EPIC) (EPIC-Italy and EPIC-IARC), and the Prostate, Lung, Colorectal, and Ovarian Cancer Screening Trial (PLCO) did not find evidence of associations with breast cancer risk [10]. In contrast, a case-cohort analysis within the Sister Study (1,566 breast cancer cases), a US cohort of women with a sister diagnosed with breast cancer, revealed associations at over 2,000 CpGs [11]. Another study with a large sample size found that genetically predicted methylation levels were associated with breast cancer risk [12], but it is unclear how predicted methylation relates to measured methylation, given that methylation varies with age and exposures accumulated over the life course [13–16].

Methylation-based markers of aging, such as Horvath-based [17], Hannum-based [18], *PhenoAge* [19] and *GrimAge* [20], have become popular tools to evaluate the association between biological aging and risk of disease. While the ‘first-generation’ measures (Horvath-based and Hannum-based) were developed to predict age accurately, *PhenoAge* and *GrimAge* are methylation-based predictors of composite measures (using clinical and physiological data) that are predictive of mortality. The residual of each of these measures on chronological age, named ‘age acceleration’, best reflects the concept of biological aging. A positive association between epigenetic aging (Horvath first-generation measure) and risk of breast cancer was first reported in an EPIC-IARC study [8], and later confirmed in the Sister Study for Horvath, Hannum and *PhenoAge* measures [21], but not for *GrimAge* [22]. Only the age acceleration based on Horvath methylation age [17] was therefore studied in relation to breast cancer risk in both previous published studies, so there is a need to accumulate evidence, particularly in women unselected for family history. Associations of epigenetic aging measures with risk of several other types of cancer were also observed in the MCCS, and these tended to be stronger for *PhenoAge* and *GrimAge* than for the first-generation measures [23, 24].

Factors other than age, mainly tobacco smoking [14, 25], alcohol consumption [15, 26] and body mass index [13, 27, 28] strongly influence blood DNA methylation and may also increase the risk of breast cancer. Similar to epigenetic aging, methylation marks of lifestyle could be useful markers to increase the precision with which we measure their association with cancer risk. These could reflect unmeasured past and cumulative exposures, imperfect assessments provided by questionnaires, or different individual responses to exposure; epigenetic predictors of lifestyle may therefore have potential to improve the prediction of breast cancer risk.

The aim of this study was to examine the association of previously derived 1) seven methylation-based measures of aging, and 2) methylation-based measures of body mass index, alcohol consumption and tobacco smoking, with breast cancer risk in a meta-analysis of individual-participant data including 1,655 breast cancer cases sampled from the MCCS, EPIC and PLCO.

## Methods

### Data sources

We used data from four methylation studies nested within three prospective cohorts of European ancestry participants: the Melbourne Collaborative Cohort Study (MCCS) [29], the European Prospective Investigation into Cancer and Nutrition (EPIC) (EPIC-Italy [7] and EPIC-IARC [8]), and the Prostate, Lung, Colorectal, and Ovarian Cancer Screening Trial (PLCO) [30]. Details about these cohorts and design of the methylation studies were described previously [10] and are provided in the Additional file 1. We used the same case selection as in our previous meta-analysis. Ductal carcinoma in situ cases were excluded from the analysis [10].

### DNA extraction, bisulphite conversion and DNA methylation data processing

Methods relating to DNA extraction and bisulphite conversion, and DNA methylation data processing have been described previously and are detailed in Additional file 1 and are the same as in our previous pooled analysis [10]. In brief, the MCCS, EPIC-Italy and EPIC-IARC measured DNA methylation using the Illumina Infinium 450 k BeadChip methylation array, and PLCO used the llumina InfiniumEPIC 850 k BeadChip methylation array. The pipeline for normalization of the methylation data was the same across the four studies (Additional file 1). β-values were calculated for each CpG site for each sample using the R package *minfi*. β-values were calculated for each CpG site for each sample using the R package *minfi*. Methylation measures with a detection *P*-value higher than 0.01 were considered missing. Samples with > 5% of CpG methylation measures missing were excluded, and CpGs with values missing for more than 20% of samples were excluded. White blood cell proportions were estimated using the Houseman algorithm modified by Jaffe and Irizarry [31, 32], using the R function *estimateCellCounts* implemented in *minfi*, or Horvath’s calculator, to derive the proportion of CD8 + T cells, CD4 + T cells, NK cells, B cells, monocytes and granulocytes.

### Methylation-based measures

#### Epigenetic aging

We used the normalised DNA methylation data to calculate the epigenetic measures of aging developed by Horvath [7] and Hannum et al. [8], as well as *PhenoAge* [9], and *GrimAge* [20] (composite biomarkers enriched for adverse phenotypes) as these have been shown to be accurate predictors of chronological age, and their deviation from chronological age (i.e. ‘age acceleration’ [AA]) was consistently found to be associated with risk of disease, cancer and death. These measures are calculated using methylation data at 353, 71, 513 and 1,030 CpGs, respectively, and were obtained using Horvath’s online calculator https://dnamage.genetics.ucla.edu/new [17, 19, 20]. Their respective age acceleration measures, defined as the residuals of the regression on chronological age, were also computed using the online calculator. Similar to other publications [21, 23], AA-Horvath and AA-Hannum measures were modified based on cell proportions. Specifically, ‘intrinsic’ epigenetic age acceleration (IEAA) is a measure of age acceleration independent of age-related changes in blood cell composition. It is computed as the residuals of the methylation age (Horvath or Hannum) on chronological age and methylation-based blood cell count estimates. ‘Extrinsic’ epigenetic age acceleration (EEAA) is computed as the residual of the Horvath methylation age on chronological age and a weighted average of age-related changes in blood cell composition. It is thought to be a measure of immune system aging. Both IEAA measures (IEAA-Horvath and IEAA-Hannum) and EEAA were estimated via the online calculator.

#### Methylation-based predictors of lifestyle

We considered a priori three established lifestyle factors associated with breast cancer risk for which there is substantial evidence of an association with DNA methylation in blood, i.e. smoking [14, 25], alcohol consumption [15, 26] and BMI [13, 27, 28]. We used the predictors by McCartney et al. [33] as these were developed and validated in a large sample of participants of mainly European ancestry (Generation Scotland) using regularised regression. The proportion of trait variance explained by these predictors was previously reported to be 61%, 12.5% and 12.5% for log of smoking pack-years, alcohol intake and BMI, respectively. Methylation predictors for BMI, smoking and alcohol consumption were calculated as the weighted average of methylation β-values at the corresponding number of CpGs, using weights available from the original publication at 1,109, 233, and 450 CpGs, respectively [33]. In each study, the methylation scores for each participant were calculated after exclusion of CpGs with missing methylation values. Each predictor was regressed on its respective risk factor—log(BMI), log(smoking pack-years) and log(alcohol consumption)—to obtain adjusted measures.

### Statistical analysis

Linear regressions between each trait and its respective epigenetic predictors were conducted to assess their association and variance explained in each predictor (Additional file 1: Table S1).

The four studies individually performed conditional (MCCS, EPIC-Italy, EPIC-IARC) or unconditional (PLCO) logistic regression to estimate the odds ratio (OR) and 95% confidence interval (95%CI) for breast cancer risk per one standard deviation (SD) increase for each of the age acceleration, smoking, alcohol intake and BMI methylation-based measures. Associations were also calculated per five-year AA increase for comparison with other studies.

Models adjusted were appropriate for the matching variables specific to each study (see Additional file 1), cell-type proportions estimated with the Houseman algorithm (percentage CD8T + , CD4T + , NK, B-cell, monocytes, granulocytes) and other variables to account for batch effects, such as plate or surrogate variable analysis (SVA) and additional adjustment for smoking (continuous pack-years), alcohol intake (continuous, grams/day) and BMI (continuous, kg/m2). Models with i) no adjustment and ii) adjustment for white blood cell proportions only, yielded very similar results and are shown in Additional file 1. Participants with missing data in any of the adjusting variables were excluded from the analysis.

Subgroup analyses within each study were carried out by conducting the same analyses (Model 1) for the following case characteristics: age at blood draw (< 50; ≥ 50 years old), time between blood draw and diagnosis (< 5; ≥ 5 years), oestrogen receptor (ER) positivity status, stage (I; II or higher).

For all analyses, estimates of pooled OR and 95%CI were calculated using fixed-effects meta-analysis, and *P*-values were calculated using the Wald test statistic. Heterogeneity in the ORs across studies was examined using the *I*^2^ statistic.

## Results

A total of 1,655 breast cancer cases were included in the analysis. The median age at blood draw was 53 years in EPIC-IARC and EPIC-Italy, 57 years in the MCCS and 62 years in PLCO. The median time from blood draw to diagnosis ranged between 6.5 years (EPIC-Italy) and 8.4 years (PLCO). Most tumours were ER positive (71% in the PLCO to 83% in EPIC samples) and diagnosed at low stage (~ 60%). For all studies, there were no large case–control differences in terms of smoking, alcohol consumption and BMI (Table 1). The description of the methylation-based predictors for each study is shown in Table 2. The range of variance explained of age by epigenetic aging measures across cohorts was: Horvath: 39% to 60%; Hannum: 48% to 64%; *PhenoAge*: 32% to 50%; *GrimAge*: 50% to 69%. The variance explained by methylation-based predictors for BMI ranged from 14 to 22%; for smoking from 41 to 54% and for alcohol consumption from 3 to 9%. All measures were strongly associated with their respective risk factor (Additional file 1: Table S1). All adjusted measures had mean 0 and standard deviation 1 and were uncorrelated with their respective variable.

**Table 1:** Characteristics of study participants

Participant characteristics	MCCS		EPIC-IARC		EPIC-Italy		PLCO	
Number of participants								
Controls	408		416		248		805	
Cases	408		416		248		583	
Age at blood draw (cases), median (IQR)	57.4 (49.8, 62.8)		53.4 (44.7–58.9)		53.6 (48.0–57.6)		62.0 (58.3, 66.2)	
Time to diagnosis, median (IQR)	7.7 (4.4, 11.1)		7.7 (5.0, 10.3)		6.5 (2.5, 10.6)		8.4 (5.6, 10.5)	
ER status, *n* (%)								
Positive	296 (73%)		347 (83%)		156 (63%)		411 (71%)	
Negative	103 (25%)		69 (17%)		32 (13%)		78 (13%)	
Missing	9 (2%)		0 (0%)		60 (24%)		94 (16%)	
Stage, *n* (%)								
I	246 (60%)		205 (49%)		71 (29%)		337 (58%)	
II or higher	140 (35%)		143 (34%)		40 (16%)		245 (42%)	
Missing	22 (5%)		68 (16%)		137 (55%)		1 (0.1%)	
Smoking, *n* (%)	Cases	Controls	Cases	Controls	Cases	Controls	Cases	Controls
Current	27 (7%)	27 (7%)	88 (21%)	94 (23%)	51 (21%)	57 (23%)	47 (8%)	51 (6%)
Former	94 (23%)	113 (28%)	87 (21%)	91 (22%)	49 (20%)	50 (20%)	203 (35%)	255 (32%)
Never	287 (70%)	268 (66%)	241 (58%)	231 (56%)	148 (60%)	141 (57%)	333 (57%)	499 (62%)
Smoking pack-years, median (IQR)	0 (0, 4.0)	0 (0, 5.7)	0 (0, 7.0)	0 (0, 6.5)	0 (0,5.1)	0 (0, 5.4)	0 (0,18.5)	0 (0,14.3)
Alcohol intake (g/day), median (IQR)	0 (0, 8.6)	0 (0, 9.5)	4.5 (0.5,16.0)	3.4 (0.4, 11.8)	2.9 (0,13.1)	2.8 (0,13.4)	0.7 (0, 7.7)	0.6 (0, 3.5)
Body mass index (kg/m^2^), median (IQR)	26 (23, 30)	26 (23, 29)	25 (23, 28)	25 (23, 28)	26 (23, 29)	25 (23, 28)	26 (23, 29)	26 (23, 30)

*MCCS* Melbourne Collaborative Cohort Study; *EPIC* European Prospective Investigation into Cancer and Nutrition; *IARC* International Agency for Research on Cancer; *PLCO* Prostate, Lung, Colorectal and Ovarian (PLCO) Cancer Screening Trial; *IQR* interquartile range; *ER* oestrogen receptor

**Table 2:** Description of methylation-based measures

Methylation-based measures	MCCS		EPIC-IARC		EPIC-Italy		PLCO	
	Median (IQR)	*R*^2,*^ (%)	Median (IQR)	*R*^2,*^ (%)	Median (IQR)	*R*^2,*^ (%)	Median (IQR)	*R*^2,*^ (%)
Horvath age	54 (47; 60)	49	51 (44; 57)	60	52 (46; 57)	64	65 (62; 69)	39
Hannum age	62 (55; 68)	59	53 (46; 60)	64	59 (53.6; 62.9)	72	56 (52; 59)	48
*PhenoAge*	54 (46; 61)	39	44 (36; 51)	50	42 (35; 48)	56	51 (47; 56)	32
*GrimAge*	53 (48; 57)	66	52 (47; 57)	69	59 (55; 63)	62	46 (42; 50)	50
BMI score	0.0 (−0.6; 0.7)	16	0.0 (−0.7; 0.7)	14	−0.1 (−0.6; 0.7)	18	0.0 (−0.7; 0.6)	22
Smoking score	−0.2 (−0.6; 0.3)	41	−0.4 (−0.6; 0.1)	44	−0.4 (−0.6; 0.2)	54	−0.3 (−0.6; 0.2)	41
Alcohol score	−0.1 (−0.6; 0.6)	3	−0.1 (−0.7; 0.6)	7	−0.1 (−0.6; 0.6)	3	−0.1 (−0.6; 0.5)	9

*MCCS* Melbourne Collaborative Cohort Study; *EPIC* European Prospective Investigation into Cancer and Nutrition; *IARC* International Agency for Research on Cancer; *PLCO* Prostate, Lung, Colorectal and Ovarian (PLCO) Cancer Screening Trial; *IQR* Interquartile range

*Variance explained of age, BMI, smoking and alcohol consumption by the corresponding methylation-based measures

Although the Horvath measures of epigenetic aging AA and IEAA were associated with risk in EPIC-IARC (similar to [8], AA-Horvath: OR per SD = 1.22, 95%CI: 1.02–1.45, IEAA-Horvath: OR = 1.22, 95%CI: 1.03–1.45), no evidence of association was found in the other cohorts (for AA: point ORs of 0.94, 0.89 and 1.03 in MCCS, EPIC-Italy, and PLCO, respectively). The pooled estimates were consistent with a null association: OR = 1.02, 95%CI: 0.95–1.10 for AA-Horvath and OR = 1.03, 95%CI: 0.96–1.11 for IEAA-Horvath after adjustment for white cell proportions, body mass index, smoking and alcohol consumption (Table 3). Similar findings were obtained for the EEAA, AA-Hannum and IEAA-Hannum measures, with pooled OR per SD = 1.02 (0.93–1.11), OR = 1.03 (0.95–1.12) and OR = 1.04 (0.96–1.12), respectively. *AA-PhenoAge* and *AA-GrimAge* age-adjusted measures also showed no association with breast cancer risk, with OR per SD: 1.01 (0.94–1.09) and OR = 1.03 (0.94–1.12), respectively), Table 3. The results for all epigenetic aging measures were virtually unchanged in models without adjustment for white blood cell proportions or lifestyle-related factors (Additional file 1: Table S2).

**Table 3:** Odds ratios^a^ for the association between methylation-based measures of breast cancer risk factors and risk of breast cancer

Methylation-based measures	MCCS	EPIC-IARC	EPIC-Italy	PLCO	*I*^2^ (%)	Pooled	*P*
	OR [95%CI]	OR [95%CI]	OR [95%CI]	OR [95%CI]		OR [95%CI]	
**Epigenetic aging**							
AA-Horvath^b^	0.95 [0.81–1.10]	1.22 [1.02–1.47]	0.89 [0.73–1.08]	1.05 [0.93–1.18]	57	**1.02 [0.95–1.10]**	0.59
IEAA-Horvath^b^	0.96 [0.83–1.10]	1.23 [1.03–1.47]	0.88 [0.72–1.06]	1.07 [0.95–1.20]	62	**1.03 [0.96–1.11]**	0.40
EEAA^b^	0.99 [0.84–1.18]	1.15 [0.94–1.41]	0.86 [0.70–1.06]	1.06 [0.92–1.22]	31	**1.02 [0.93–1.11]**	0.67
AA-Hannum^b^	0.97 [0.83–1.14]	1.19 [0.97–1.45]	0.86 [0.70–1.06]	1.09 [0.96–1.24]	51	**1.03 [0.95–1.12]**	0.43
IEAA-Hannum^b^	0.98 [0.84–1.13]	1.18 [0.98–1.42]	0.88 [0.72–1.08]	1.08 [0.96–1.22]	45	**1.04 [0.96–1.12]**	0.34
*PhenoAge*	0.97 [0.83–1.14]	1.12 [0.94–1.34]	0.89 [0.73–1.10]	1.03 [0.91–1.17]	4	**1.01 [0.94–1.09]**	0.75
*GrimAge*	1.03 [0.86–1.25]	1.08 [0.90–1.30]	0.94 [0.79–1.13]	1.05 [0.91–1.21]	0	**1.03 [0.94–1.12]**	0.53
**Lifestyle-related factors**							
BMI methylation score	1.17 [1.01–1.36]	1.06 [0.92–1.23]	1.25 [1.03–1.52]	1.02 [0.91–1.15]	26	**1.10 [1.02–1.18]**	0.01
Adjusted BMI score^c^	1.11 [0.96–1.28]	1.02 [0.88–1.17]	1.27 [1.04–1.55]	1.06 [0.94–1.19]	12	**1.09 [1.01–1.17]**	0.02
Smoking methylation score	0.98 [0.84–1.14]	0.95 [0.82–1.11]	1.11 [0.90–1.35]	1.13 [1.00–1.27]	24	**1.04 [0.97–1.12]**	0.25
Adjusted smoking score^c^	1.01 [0.87–1.17]	0.94 [0.81–1.09]	1.13 [0.94–1.34]	1.08 [0.97–1.22]	6	**1.04 [0.97–1.12]**	0.29
Alcohol methylation score	0.95 [0.82–1.11]	1.01 [0.87–1.17]	0.83 [0.67–1.03]	1.07 [0.95–1.20]	32	**1.00 [0.93–1.07]**	0.91
Adjusted alcohol score^c^	0.95 [0.82–1.11]	0.96 [0.83–1.12]	0.85 [0.69–1.05]	1.03 [0.91–1.16]	0	**0.97 [0.90–1.04]**	0.42

Bold values indicate results pooled across the four studies

^a^Adjusting for white blood cell proportions, BMI, smoking and alcohol consumption

^b^These models were not adjusted for white blood cell proportions because these are integrated in the IEAA and EEAA measures

^c^Residuals from the regression of the scores on the log of their respective lifestyle variables

Associations for methylation-based predictors of lifestyle-related factors are shown in Table 3. The predictor of BMI was positively associated with breast cancer risk in the pooled analysis with an OR of 1.10 per SD (95%CI: 1.02–1.18), with little heterogeneity across cohorts (*I*^2^ = 26%). This association was virtually not attenuated when considering the adjusted measure, i.e. BMI predictor regressed on BMI: OR = 1.09, 95%CI: 1.01–1.17 (*I*^2^ = 12%). Estimates were similar in unadjusted models (Additional file 1: Table S2). There was limited evidence that the methylation-based predictors of smoking (OR = 1.04, *P* > 0.05) and alcohol consumption (OR = 1.0, *P* > 0.05), or their respective adjusted measures, were associated with breast cancer risk (Table 3).

None of the associations of epigenetic aging or lifestyle measures with breast cancer risk showed substantial heterogeneity by age at blood draw, time to diagnosis, ER positivity or tumour stage at diagnosis (Table 4); for *AA-GrimAge*, the association appeared stronger for ER-negative cases (OR = 1.18, 95%CI: 1.00–1.40). The adjusted BMI measures appeared somewhat more strongly associated with risk for women diagnosed within five years from blood draw (OR = 1.15, 95%CI: 1.01–1.32) compared with those diagnosed more than five years after blood draw (OR = 1.08, 95%CI: 1.00–1.17).

**Table 4:** Odds ratios^a^ (pooled analysis) for subgroup analyses of the association between methylation-based measures of aging and lifestyle (regressed on their respective risk factors) and risk of breast cancer (*N* = 1,655)

	AA-Horvath^b^ OR [95%CI]	AA-Hannum^b^ OR [95%CI]	*PhenoAge*^b^ OR [95%CI]	*GrimAge*^b^ OR [95%CI]	Adj. BMI score^c^ OR [95%CI]	Adj. smoking score^c^ OR [95%CI]	Adj. alcohol score^c^ OR [95%CI]
Time to diagnosis							
< 5 years	1.01 [0.88–1.16]	0.98 [0.84–1.13]	1.01 [0.88–1.17]	1.04 [0.92–1.19]	1.15 [1.01–1.32]	0.99 [0.87–1.13]	1.01 [0.88–1.15]
≥ 5 years	1.02 [0.94–1.11]	1.06 [0.97–1.16]	1.03 [0.95–1.13]	1.05 [0.96–1.14]	1.08 [1.00–1.17]	1.06 [0.98–1.14]	0.96 [0.88–1.05]
Age at blood draw							
< 50	1.12 [0.92–1.36]	1.08 [0.89–1.32]	1.02 [0.84–1.23]	1.10 [0.93–1.30]	1.10 [0.93–1.30]	0.99 [0.85–1.14]	0.96 [0.81–1.14]
≥ 50	1.00 [0.92–1.09]	1.03 [0.94–1.12]	1.03 [0.95–1.12]	1.03 [0.95–1.11]	1.10 [1.02–1.19]	1.04 [0.96–1.13]	0.96 [0.89–1.05]
ER positivity status							
ER positive	1.06 [0.98–1.16]	1.04 [0.95–1.14]	1.01 [0.93–1.10]	1.03 [0.95–1.12]	1.05 [0.97–1.14]	1.03 [0.95–1.12]	0.97 [0.89–1.06]
ER negative	0.87 [0.73–1.04]	0.93 [0.77–1.12]	1.02 [0.85–1.23]	1.18 [1.00–1.40]	1.15 [0.97–1.37]	1.05 [0.89–1.23]	0.92 [0.76–1.10]
Stage							
I	1.05 [0.95–1.16]	1.04 [0.94–1.16]	1.02 [0.92–1.12]	1.07 [0.98–1.18]	1.09 [0.99–1.19]	1.03 [0.94–1.14]	0.96 [0.87–1.06]
II or higher	1.00 [0.88–1.15]	1.08 [0.93–1.25]	1.04 [0.91–1.20]	1.08 [0.94–1.24]	1.08 [0.95–1.23]	1.12 [0.98–1.28]	1.02 [0.89–1.17]

^a^Adjusting for white blood cell proportions, except for AA-Horvath and AA-Hannum

^b^Regressed on age

^**c**^Regressed on log(BMI), log(pack-years) and log(alcohol in grams/day), respectively

## Discussion

We have assessed seven measures of epigenetic aging and three methylation-based predictors of lifestyle for their association with breast cancer risk in a large sample (1,655 cases) of women from Western countries (Australia, Europe and the USA). We found overall no associations between measures of epigenetic aging and risk of breast cancer. A positive association was observed for the BMI methylation score, but not for smoking and alcohol consumption.

To our knowledge few studies have investigated the association of epigenetic aging with breast cancer risk. We included in this meta-analysis the samples for which an association was reported previously in EPIC-IARC [8]. Slightly different models were used but the results were very similar. The association previously observed in EPIC-IARC was restricted to postmenopausal women (per 1-year IEAA-Horvath: OR = 1.06, 95%CI: 1.02–1.11) compared with OR = 1.00 for premenopausal women. We found no evidence of an association in our meta-analysis, including when restricted to ages older than 50 years. Our results are overall consistent with the findings from the Sister Study [34], which reported relatively weak associations: based on 1,566 cases, per 5-year AA-Hannum: hazard ratio (HR) = 1.10, 95%CI, 1.00–1.21, AA-Horvath: HR = 1.08, 95%CI = 1.00–1.17, and *AA-PhenoAge*: HR = 1.15, 95%CI = 1.07–1.23. In our study, the ORs expressed per 5-year AA were compatible for AA-Hannum and AA-Horvath (HR = 1.02, 95%CI, 0.94–1.10 and HR = 1.01, 95%CI = 0.94–1.08, respectively) and more discrepant for *AA-PhenoAge*: HR = 1.00, 95%CI = 0.95–1.06. Similar to our findings, the authors did not find substantial heterogeneity by, for example, menopausal or ER-positivity status. For *AA-GrimAge*, the authors expressed the association per standard deviation [22] and found HR = 1.06, 95%CI: 0.98, 1.14, which is also similar to our study OR = 1.03, 95%CI: 0.94–1.12. Although *AA-GrimAge* appeared somewhat more strongly associated with risk for postmenopausal women in the Sister Study, the evidence for heterogeneity was weak and there was no indication of this in our data (HR = 1.03 for women aged ≥ 50 years at blood draw).

The main differences between the cohorts included in our meta-analysis and the Sister Study are that it was enriched for family history of breast cancer and had substantially shorter length of follow-up than ours (for the cases, mean time to diagnosis of 3.9 years, compared with > 6 years for all studies we included). We nevertheless did not observe that OR estimates were larger when blood was collected closer to diagnosis (within 5 years: OR ~ 1.01, 0.98, 1.01, 1.04 for AA-Horvath, AA-Hannum, *AA-PhenoAge* and *AA-GrimAge*, respectively). The study of Durso and colleagues [35] compared Horvath and Hannum age acceleration measures between 233 Italian women who developed breast cancer (mean age at recruitment: 52.4 years, mean time to diagnosis: 3.8 years) and cancer-free controls and found no evidence of an association. A study of multiple health outcomes using Generation Scotland data included 83 incident breast cancer cases, diagnosed over 13 years of follow-up in women aged ~ 51 years at baseline [36]. A tendency for risk associations to be positive was observed: per SD, AA-Horvath, HR = 1.01 (*P* = 0.95), AA-Hannum: HR = 1.24 (*P* = 0.07), *AA-PhenoAge*: HR = 1.36 (*P* = 0.01), and *AA-GrimAge* = 1.19 (*P* = 0.16), respectively, in age-adjusted models. The literature to date therefore includes, to our knowledge, approximately 3,550 breast cancer cases and is consistent with a weak (of roughly 8% increase per 5-year AA for *AA-PhenoAge*) or null association between epigenetic aging measured in blood and breast cancer risk.

There has not been to our knowledge any study examining methylation-based predictors of lifestyle-related factors with risk of breast cancer. A handful of studies have examined risk of overall mortality [33], survival from oropharyngeal cancer [37] and risk of several types of cancer in the Melbourne Collaborative Cohort Study [38]. Another study used the Cancer Genome Atlas datasets to develop lifestyle predictors based on tumour DNA methylation [39] and found that the BMI-associated methylation signature was predictive of shorter breast cancer survival. For the methylation-based predictors used in our study, the variance explained was somewhat higher than that originally reported by McCartney et al. [33] for BMI (12%), but somewhat lower for smoking and alcohol consumption (61% and 12%, respectively). For smoking, it may be because it was trained to predict log (pack-years) in current smokers, and our analysis also included former smokers; analyses of the MCCS data showed that the *R*^2^ was 66% when former smokers were excluded (not shown). Other methylation-based measures of lifestyle have been developed showing similar accuracy, e.g. for alcohol [26], or smoking [25, 40], and were not tested in the current study; we chose to use these predictors because they were developed using a large sample size of people of similar ancestry (Scottish) and were well validated. In MCCS analyses of other cancer types, the choice of predictor did not appear to make a substantial difference in the observed associations [38]. In another analysis of the Sister Study data, the authors used as inputs to predict breast cancer risk 36 methylation-based measures of biological aging and physiological characteristics and methylation values at 100 individual CpGs (i.e. using altogether methylation values at thousands of CpGs) and derived a risk score that showed reasonable performance with an area under the curve of 0.63, which was similar to, and independent of, the association observed for the 313-SNP polygenic risk score [41]. We did not attempt to combine methylation scores in our study because most associations were weak, but it is likely that this type of approach may yield improvements to breast cancer risk prediction in the future.

That we observed only weak or null associations may be explained by the fact that none of BMI, alcohol consumption or smoking are strong risk factors for breast cancer. Previous studies have generally found weak to moderate associations [33, 37, 38], except for lung cancer in the MCCS [38], for which the effect of smoking is dramatic. We had hypothesised that methylation predictors of BMI, alcohol and smoking could contain more information about lifestyle than the measured risk factors—for example, exposures accumulated over the lifetime, in particular, during sensitive periods such as early life or the periconceptional period, which could be better captured by DNA methylation compared with questionnaires at older ages. BMI has consistently been found to be positively associated with risk of breast cancer for postmenopausal women and negatively for pre-menopausal women [42]; we did not observe this using methylation scores as the estimates of associations were similar by age at blood draw (< 50 years: HR = 1.10 [0.93–1.30] and ≥ 50 years: HR = 1.10 [1.02–1.19]). The association we observed for BMI might also reflect the combined effect of several aspects of obesity beyond BMI [43] that could be captured by changes in DNA methylation. For other breast cancer risk factors, there is to date no convincing evidence that they are strongly associated with blood DNA methylation changes, e.g. for mammographic density [44] or lifetime oestrogen exposure [45]. Additional risk factors for breast cancer were not adjusted for, but this would probably make little difference to the results given their confounding effect on the lifestyle methylation-breast cancer association is likely small.

The main strength of our study is the largest sample size to date of ~ 1,650 cases with long follow-up and comprehensive assessment of epigenetic measures for association with risk. The same analysis method was applied across cohort datasets and participants were representative of the general population. Limitations of our study include the relative heterogeneity of the pooled samples; even though most participants were of European ancestry, there was some variation in terms of age at inclusion, follow-up time or sample processing. All studies used the same pipeline for normalisation of the data, but PLCO used the EPIC assay, which may result in small measurement differences.

## Conclusion

Our study found overall weak associations of methylation-based measures of aging and lifestyle-related with breast cancer risk. The association observed for a BMI methylation score might provide insights in the underlying association between BMI and breast cancer and should be further investigated in additional studies.

## Supplementary Information

**Additional file 1.** Supplementary Methods and Tables.

## Data Availability

The datasets generated during and/or analysed during the current study are available from the corresponding author on reasonable request.
