# Supplementary material for: Methylation-based markers of aging and lifestyle-related factors and risk of breast cancer: a pooled analysis of four prospective studies
Source: Breast Cancer Res. 2022 Sep 6;24:59. doi: 10.1186/s13058-022-01554-8 (PMC9446544; doi:10.1186/s13058-022-01554-8)
Supplement: Supplementary file 1 — Additional file 1. Supplementary Methods and Tables. [file 13058_2022_1554_MOESM1_ESM.docx]

**Supplementary material for “Methylation-based measures of aging and lifestyle and risk of breast cancer: A pooled analysis of four prospective studies”**

**Description of cohort and methylation studies**

*Melbourne Collaborative Cohort Study (MCCS)*

The Melbourne Collaborative Cohort Study (MCCS) is a prospective cohort study that included 41,513 adult women (24,469; 59%) and men (17,045; 41%) aged 40–69 years (for 99% of them) at baseline. Participants were recruited from the Melbourne metropolitan area between 1990 and 1994, at which time information on demographics, lifestyle factors and medical conditions was collected through questionnaires, and clinical measurements were taken by trained staff. Greek-born and Italian-born participants were oversampled to extend the range of dietary, lifestyle and genetic characteristics of the population **[1]**. Cases (N=409) in the nested case-control study were women with a first diagnosis of invasive adenocarcinoma of the breast (International Classification of Diseases for Oncology, C50) occurring between baseline interview and 31 December 2007, as described in Severi et al. **[2]**. Cases were ascertained by record linkage to the population-based Victorian cancer registry (VCR), and to the Australian Cancer Database. Controls (N=409) were selected through density sampling and matched with cases on year of birth, year of baseline attendance, country of origin and, when possible, type of baseline blood specimen (dried blood spot, buffy coat or lymphocyte). Only case-control pairs with matching sample type were included in this analysis, excluding 11 case-control pairs from the 420 pairs used in the Severi *et al.* study **[2]**. A cancer case was considered early stage if it was diagnosed with stage I and it was considered late stage if it was diagnosed with stage II, III or IV (using the definition from the AJCC 7^th^ edition manual).

*European Prospective Investigation into Cancer and Nutrition (EPIC-Italy)*

Participants for this nested case-control study were selected from the Italian cohort of the European Prospective Investigation into Cancer and Nutrition (EPIC) study **[3]**. This sub-cohort consists of 46,857 volunteers (including 32,157 women), recruited from 5 different centers within Italy (Varese, Turin, Florence, Naples, and Ragusa). Incident cases were identified through cancer registries with <2 % losses to follow-up. We identified 248 incident female breast cancer cases, for each of which we collected 248 healthy female controls (matched on date of birth (±5 years), year of recruitment and study center). Average follow-up (cases and controls combined) was 180.4 months (range: 4.0–215.4) and average time to diagnosis was 85.5 months (range 0.53–211.23). For all study participants, detailed baseline information about lifestyle habits and personal and family history was collected through questionnaires, along with blood samples and anthropometric measurements at enrolment between 1993 and 1998. A cancer case was considered early stage if it was classified as “localized” while it was considered late stage if it was classified as “regional” or “metastatic”. All participants signed an informed consent form, and the ethical review boards of the International Agency for Research on Cancer (IARC) and of local participating centers approved the study protocol.

*European Prospective Investigation into Cancer and Nutrition (EPIC-IARC)*

Participants for this nested case-control study were selected from the IARC cohort of the EPIC study **[4]** and included subjects from 6 different countries: Germany, Greece, Italy (non-overlapping to EPIC-Italy), Spain, The Netherlands and UK. Incident cases were identified at regular intervals through population-based cancer registries (Italy, Spain, The Netherlands and UK) or active follow-up (Germany and Greece), which included a review of health insurance records, cancer and pathology registries, and direct contact with participants and their next-of-kin. The analytic nested case-control study consisted of 416 incident female breast cancer cases and 416 matched healthy female controls, matched on center, age, date and time of blood collection, fasting status, menopause, current pill and hormone replacement therapy use and menstrual cycle. For all study participants, detailed baseline information about lifestyle habits and personal and family history was collected through questionnaires, along with blood samples and anthropometric measurements at enrolment between 1993 and 1998. A cancer case was considered early stage if it was classified as “localized” while it was considered late stage if it was classified as “regional” or “metastatic”. All participants signed an informed consent form, and the ethical review boards of the International Agency for Research on Cancer (IARC) and of local participating centers approved the study protocol.

*Prostate, Lung, Colorectal and Ovarian screening trial (PLCO)*

The Prostate, Lung, Colorectal, and Ovarian Cancer Screening Trial (PLCO) is a population-based multicenter randomized screening trial of people age 55 to 74 years at baseline with no history of prostate, lung, colorectal, or ovarian cancer (NCT00339495) **[5]**. This study was approved by institutional review boards at the US National Cancer Institute and the 10 centers. Our nested case–control were selected from cases and controls previously selected for the OncoArray project **[6, 7]** with blood samples (buffy coat) available at the time of study entry. It included 583 incident invasive primary breast cancer cases (ICD-9 174.0-174.9). The 805 controls were frequency matched on age at random assignment (5-year intervals), and fiscal year of randomization (pre/post 10/1/1997). All controls were alive and had no history of cancer as of the date of diagnosis for the matched case. A cancer case was considered early stage if it was diagnosed with stage I and it was considered late stage if it was diagnosed with stage II, III or IV (using the definition from the AJCC 5^th^ edition manual).

**REFERENCES**

1. Milne RL, Fletcher AS, MacInnis RJ, Hodge AM, Hopkins AH, Bassett JK, Bruinsma FJ, Lynch BM, Dugue PA, Jayasekara H *et al*: **Cohort Profile: The Melbourne Collaborative Cohort Study (Health 2020)**. *International journal of epidemiology* 2017, **46**(6):1757-1757i.

2. Severi G, Southey MC, English DR, Jung C-h, Lonie A, McLean C, Tsimiklis H, Hopper JL, Giles GG, Baglietto L: **Epigenome-wide methylation in DNA from peripheral blood as a marker of risk for breast cancer**. *Breast cancer research and treatment* 2014, **148**(3):665-673.

3. van Veldhoven K, Polidoro S, Baglietto L, Severi G, Sacerdote C, Panico S, Mattiello A, Palli D, Masala G, Krogh V *et al*: **Epigenome-wide association study reveals decreased average methylation levels years before breast cancer diagnosis**. *Clinical Epigenetics* 2015, **7**(1):67.

4. Ambatipudi S, Horvath S, Perrier F, Cuenin C, Hernandez-Vargas H, Le Calvez-Kelm F, Durand G, Byrnes G, Ferrari P, Bouaoun L *et al*: **DNA methylome analysis identifies accelerated epigenetic ageing associated with postmenopausal breast cancer susceptibility**. *European Journal of Cancer* 2017, **75**:299-307.

5. Prorok PC, Andriole GL, Bresalier RS, Buys SS, Chia D, Crawford ED, Fogel R, Gelmann EP, Gilbert F, Hasson MA *et al*: **Design of the Prostate, Lung, Colorectal and Ovarian (PLCO) Cancer Screening Trial**. *Controlled clinical trials* 2000, **21**(6 Suppl):273S-309S.

6. Amos CI, Dennis J, Wang Z, Byun J, Schumacher FR, Gayther SA, Casey G, Hunter DJ, Sellers TA, Gruber SB *et al*: **The OncoArray Consortium: A Network for Understanding the Genetic Architecture of Common Cancers**. *Cancer epidemiology, biomarkers & prevention : a publication of the American Association for Cancer Research, cosponsored by the American Society of Preventive Oncology* 2017, **26**(1):126-135.

7. Michailidou K, Lindstrom S, Dennis J, Beesley J, Hui S, Kar S, Lemacon A, Soucy P, Glubb D, Rostamianfar A *et al*: **Association analysis identifies 65 new breast cancer risk loci**. *Nature* 2017, **551**(7678):92-94.

**Supplementary Table 1.** Association between methylation-based predictors and their respective risk factor.

| **Cohort** | **Methylation-based measure** | **Regression coef.^a^** | **SE(Coef.)** | **P** | **R^2^** |
| --- | --- | --- | --- | --- | --- |
| **MCCS** | log (BMI +1) | 0.08 | 0.007 | 1.3E-32 | 0.16 |
|  | Current smoker | 2.85 | 0.098 | 7.4E-128 | 0.53 |
|  | Former smoker | 0.68 | 0.056 | 2.2E-31 |  |
|  | log (pack-years +1) | 0.46 | 0.020 | 2.1E-94 | 0.41 |
|  | log (alcohol +1) | 0.13 | 0.027 | 1.4E-06 | 0.03 |
|  | Horvath age | 0.83 | 0.030 | 6.5E-123 | 0.49 |
|  | Hannum age | 0.86 | 0.025 | 5.4E-162 | 0.59 |
|  | PhenoAge | 0.83 | 0.037 | 4.8E-88 | 0.38 |
|  | GrimAge | 0.70 | 0.018 | 9.9E-193 | 0.66 |
| **EPIC-IARC** | log (BMI +1) | 2.31 | 0.203 | 5.0E-28 | 0.14 |
|  | Current smoker | 1.80 | 0.061 | 1.0E-131 | 0.51 |
|  | Former smoker | 0.38 | 0.061 | 7.9E-10 |  |
|  | log (pack-years +1) | 0.53 | 0.021 | 1.6E-106 | 0.44 |
|  | log (alcohol +1) | 0.22 | 0.027 | 1.3E-15 | 0.07 |
|  | Horvath age | 0.77 | 0.022 | 5.5E-169 | 0.60 |
|  | Hannum age | 0.79 | 0.021 | 1.3E-185 | 0.64 |
|  | PhenoAge | 0.83 | 0.029 | 3.8E-128 | 0.50 |
|  | GrimAge | 0.68 | 0.016 | 1.2E-214 | 0.69 |
| **EPIC-Italy** | log (BMI +1) | 0.09 | 0.009 | 2.2E-24 | 0.18 |
|  | Current smoker | 0.41 | 0.075 | 8.5E-08 | 0.56 |
|  | Former smoker | 1.86 | 0.072 | 1.9E-94 |  |
|  | log (pack-years +1) | 0.69 | 0.027 | 6.6E-91 | 0.54 |
|  | log (alcohol +1) | 0.13 | 0.033 | 6.1E-05 | 0.03 |
|  | Horvath age | 0.03 | 0.046 | 4.6E-01 | 0.64 |
|  | Hannum age | 0.05 | 0.042 | 2.1E-01 | 0.72 |
|  | PhenoAge | 0.08 | 0.056 | 1.4E-01 | 0.56 |
|  | GrimAge | 0.64 | 0.022 | 7.4E-111 | 0.62 |
| **PLCO** | log (BMI +1) | 2.71 | 0.136 | 2.1E-77 | 0.22 |
|  | Current smoker | 2.88 | 0.071 | 5.4E-237 | 0.56 |
|  | Former smoker | 0.67 | 0.039 | 5.4E-60 |  |
|  | log (pack-years +1) | 0.41 | 0.013 | 2.1E-161 | 0.41 |
|  | log (alcohol +1) | 0.26 | 0.022 | 1.7E-29 | 0.09 |
|  | Horvath age | 0.67 | 0.022 | 4.1E-152 | 0.39 |
|  | Hannum age | 0.77 | 0.022 | 4.1E-200 | 0.48 |
|  | PhenoAge | 0.80 | 0.032 | 1.1E-116 | 0.32 |
|  | GrimAge | 0.74 | 0.020 | 1.9E-211 | 0.50 |

**^a^** Univariate linear regression models

**Supplementary Table 2.** Odds ratios for the association between methylation-based measures of breast cancer risk factors and risk of breast cancer.

| **Methylation-based measures** |  | **MCCS** | **EPIC-IARC** | **EPIC-Italy** | **PLCO** | **Pooled** | **P** |
| --- | --- | --- | --- | --- | --- | --- | --- |
| **Epigenetic aging** |  | OR [95%CI] | OR [95%CI] | OR [95%CI] | OR [95%CI] | **OR [95%CI]** |  |
| **AA-Horvath** | Model 0^a^ | 0.91 [0.79-1.05] | 1.17 [0.99-1.40] | 0.90 [0.74-1.08] | 1.02 [0.91-1.14] | **1.00 [0.93-1.07]** | 0.93 |
|  | Model 1 ^a^ | 0.95 [0.81-1.10] | 1.22 [1.02-1.47] | 0.89 [0.73-1.08] | 1.05 [0.93-1.18] | **1.02 [0.95-1.10]** | 0.59 |
| **IEAA-Horvath** | Model 0 | 0.94 [0.82-1.08] | 1.22 [1.03-1.45] | 0.89 [0.74-1.07] | 1.05 [0.94-1.18] | **1.02 [0.95-1.10]** | 0.54 |
|  | Model 1 | 0.96 [0.83-1.10] | 1.23 [1.03-1.47] | 0.88 [0.72-1.06] | 1.07 [0.95-1.20] | **1.03 [0.96-1.11]** | 0.40 |
| **EEAA** | Model 0 | 0.98 [0.85-1.13] | 1.10 [0.93-1.30] | 0.87 [0.72-1.06] | 1.03 [0.92-1.15] | **1.01 [0.94-1.08]** | 0.87 |
|  | Model 1 | 0.99 [0.84-1.18] | 1.15 [0.94-1.41] | 0.86 [0.70-1.06] | 1.06 [0.92-1.22] | **1.02 [0.93-1.11]** | 0.67 |
| **AA-Hannum** | Model 0 | 0.96 [0.84-1.11] | 1.15 [0.96-1.36] | 0.87 [0.72-1.07] | 1.06 [0.94-1.18] | **1.02 [0.95-1.10]** | 0.63 |
|  | Model 1 | 0.97 [0.83-1.14] | 1.19 [0.97-1.45] | 0.86 [0.70-1.06] | 1.09 [0.96-1.24] | **1.03 [0.95-1.12]** | 0.43 |
| **IEAA-Hannum** | Model 0 | 0.98 [0.85-1.13] | 1.18 [0.99-1.41] | 0.90 [0.74-1.09] | 1.07 [0.95-1.19] | **1.04 [0.96-1.11]** | 0.34 |
|  | Model 1 | 0.98 [0.84-1.13] | 1.18 [0.98-1.42] | 0.88 [0.72-1.08] | 1.08 [0.96-1.22] | **1.04 [0.96-1.12]** | 0.34 |
| ***PhenoAge*** | Model 0 | 0.95 [0.83-1.09] | 1.11 [0.95-1.31] | 0.90 [0.74-1.10] | 1.04 [0.93-1.16] | **1.01 [0.94-1.09]** | 0.74 |
|  | Model 1 | 0.97 [0.83-1.14] | 1.12 [0.94-1.34] | 0.89 [0.73-1.10] | 1.03 [0.91-1.17] | **1.01 [0.94-1.09]** | 0.75 |
| ***GrimAge*** | Model 0 | 0.99 [0.86-1.14] | 1.04 [0.91-1.20] | 0.93 [0.78-1.11] | 1.11 [0.99-1.24] | **1.04 [0.97-1.11]** | 0.29 |
|  | Model 1 | 1.03 [0.86-1.25] | 1.08 [0.90-1.30] | 0.94 [0.79-1.13] | 1.05 [0.91-1.21] | **1.03 [0.94-1.12]** | 0.53 |
| **Lifestyle factors** |  |  |  |  |  |  |  |
| **BMI methylation score** | Model 0 | 1.13 [0.99-1.30] | 1.07 [0.94-1.23] | 1.23 [1.02-1.48] | 1.04 [0.93-1.16] | **1.09 [1.02-1.17]** | 0.01 |
|  | Model 1 | 1.17 [1.01-1.36] | 1.06 [0.92-1.23] | 1.25 [1.03-1.52] | 1.02 [0.91-1.15] | **1.10 [1.02-1.18]** | 0.01 |
| **Adjusted BMI score^b^** | Model 0 | 1.06 [0.93-1.22] | 1.03 [0.90-1.18] | 1.23 [1.02-1.49] | 1.08 [0.97-1.21] | **1.08 [1.01-1.16]** | 0.02 |
|  | Model 1 | 1.11 [0.96-1.28] | 1.02 [0.88-1.17] | 1.27 [1.04-1.55] | 1.06 [0.94-1.19] | **1.09 [1.01-1.17]** | 0.02 |
| **Smoking methylation score** | Model 0 | 0.99 [0.86-1.15] | 1.00 [0.87-1.15] | 1.06 [0.88-1.28] | 1.14 [1.02-1.27] | **1.06 [0.99-1.13]** | 0.10 |
|  | Model 1 | 0.98 [0.84-1.14] | 0.95 [0.82-1.11] | 1.11 [0.90-1.35] | 1.13 [1.00-1.27] | **1.04 [0.97-1.12]** | 0.25 |
| **Adjusted smoking score^b^** | Model 0 | 1.02 [0.88-1.17] | 0.95 [0.83-1.10] | 1.11 [0.93-1.31] | 1.08 [0.97-1.20] | **1.04 [0.97-1.11]** | 0.28 |
|  | Model 1 | 1.01 [0.87-1.17] | 0.94 [0.81-1.09] | 1.13 [0.94-1.34] | 1.08 [0.97-1.22] | **1.04 [0.97-1.12]** | 0.29 |
| **Alcohol methylation score** | Model 0 | 0.97 [0.85-1.12] | 0.99 [0.86-1.13] | 0.82 [0.68-1.00] | 1.10 [0.99-1.23] | **1.00 [0.94-1.07]** | 0.92 |
|  | Model 1 | 0.95 [0.82-1.11] | 1.01 [0.87-1.17] | 0.83 [0.67-1.03] | 1.07 [0.95-1.20] | **1.00 [0.93-1.07]** | 0.91 |
| **Adjusted alcohol score^b^** | Model 0 | 0.98 [0.85-1.12] | 0.95 [0.83-1.09] | 0.83 [0.69-1.01] | 1.05 [0.94-1.18] | **0.98 [0.91-1.05]** | 0.55 |
|  | Model 1 | 0.95 [0.82-1.11] | 0.96 [0.83-1.12] | 0.85 [0.69-1.05] | 1.03 [0.91-1.16] | **0.97 [0.90-1.04]** | 0.42 |

^a^ Model 0. No adjustment; Model 1. Adjusted for white blood cell proportions

^b^ Residuals from the regression of the score on the log of their respective lifestyle variable
